# Return of secondary findings in genomic sequencing: Military implications

**DOI:** 10.1002/mgg3.483

**Published:** 2018-11-10

**Authors:** Lydia D. Hellwig, Clesson Turner, Teri A. Manolio, Mark Haigney, Cynthia A. James, Brittney Murray, Dale F. Szpisjak, Sheila Muldoon, Juvianee Estrada‐Veras, Alyson Krokosky, Mauricio J. De Castro

**Affiliations:** ^1^ The Henry M. Jackson Foundation for the Advancement of Military Medicine, Inc Bethesda Maryland; ^2^ Uniformed Services University of the Health Sciences Bethesda Maryland; ^3^ Walter Reed National Military Medical Center Bethesda Maryland; ^4^ National Human Genome Research Institute Bethesda Maryland; ^5^ Department of Medicine Uniformed Services University of the Health Sciences Bethesda Maryland; ^6^ Department of Medicine, Johns Hopkins School of Medicine Johns Hopkins University Baltimore Maryland; ^7^ Division of Cardiology Johns Hopkins University School of Medicine Baltimore Maryland; ^8^ Department of Anesthesiology Uniformed Services University of the Health Sciences Bethesda Maryland; ^9^ 81st Medical Group Air Force Medical Genetics Center Biloxi Mississippi

**Keywords:** genomics, military, secondary findings

## Abstract

**Background:**

Genomic sequencing has become a widely used tool in clinical and research settings in both civilian and military healthcare systems.

**Methods:**

In this paper, we consider potential military‐specific implications of returning genomic sequencing secondary findings to ensure the proper protections, policies, and processes are in place for the use of this information.

**Results:**

We specifically use two examples to highlight potential military implications of the return of secondary findings.

**Conclusion:**

Clinicians and researchers are strongly encouraged to consider the military implications of the return of results for informed consent of service members or their families undergoing clinical or research genomic sequencing.

## 1 INTRODUCTION

Genomic sequencing initiatives are essential to advancing genomic medicine. There is a limited body of evidence on the military‐specific implications of results returned from these studies, in particular as it pertains to secondary findings (Castro & Turner, 2017; Kruszka, Weiss, & Hadley, 2017). We highlight potential military implications of the return of secondary findings using two example conditions, and strongly encourage clinicians and researchers to consider these for proper informed consent of service members or their families undergoing clinical or research genomic sequencing.

## 2 GENETIC DISEASE AND THE MILITARY

Protections against genetic discrimination differ in the military and civilian populations. Although the Genetic Information Nondiscrimination Act of 2008 does not cover Active Duty Service Members (SMs), there are military‐specific policies in place governing how genetic information is managed in the military healthcare system (Castro et al., 2016). A SM's health status directly impacts their ability to successfully carry out their mission, with potential national security and defense ramifications. It follows that certain health conditions, both genetic and non‐genetic, disqualify an individual from serving in the military, and these conditions may vary by branch of service or specific duty. These conditions may disqualify an individual from enlisting, render a SM unfit to continue to serve on active duty, and impact eligibility for disability compensation. Each branch has its own evaluation processes for determining whether or not a SM's medical condition enables them to continue to meet medical retention standards.

For a genetic disorder to become a disqualifying condition, this disorder must impact their ability to perform specific duties or be deployed worldwide. In the great majority of cases, this means that the individual must have developed a phenotype that is severe enough to impact their ability to perform their duty. With some important exceptions, genetic variants identified through genomic sequencing should not result in any action unless that individual suffers symptoms during their time of service and those symptoms limit the individual's ability to perform their duties.

## 3 SECONDARY FINDINGS

The American College of Medical Genetics and Genomics (ACMG) and the Association for Molecular Pathology (AMP) guidelines recommend that pathogenic and likely pathogenic findings in 59 clinically actionable genes, unrelated to the underlying reason for testing, be returned by clinical laboratories for clinicians to use, with appropriate consent, in their patients’ care (Kalia et al., 2016). Although developed for clinical testing, many research initiatives use these 59 genes as a starting point for return of secondary findings. Studies have shown that participants in genomic sequencing studies expect and desire to learn about clinically relevant genomic results (Faucett & Davis, 2016). Returnable findings in these genes are anticipated to be found in approximately 3%–5% of individuals undergoing genomic sequencing (Dewey et al., 2016). It is important to note that the hereditary conditions related to the genes on the ACMG Secondary Findings v2.0 list show incomplete penetrance and variable expressivity. Therefore, it is possible that individuals with pathogenic or likely pathogenic variants in these genes may never develop symptoms.

## 4 POTENTIAL CAREER IMPACT OF SECONDARY FINDINGS

As discussed above, in most cases, genetic variation does not equate to disease or disability. In these conditions, such as for a patient discovered to have a pathogenic variant in* BRCA1*, the presence of the genetic finding alone should not impact their military career, even if the finding prompts a need for follow‐up or risk‐reduction surgical interventions. If in the course of follow‐up a diagnosis is made, this may impact service ability, but only if the clinical manifestations of the condition render the SM unfit for duty.

The majority of the genes on ACMG 59 gene list are related to conditions that would not be expected to affect continued service in asymptomatic individuals. However, pathogenic variants identified in certain genes listed could have potential implications in ostensibly healthy individuals. This is due to the nature of the genes involved, the potential sudden clinical presentation, and associated medical management guidelines for those who have a pathogenic variant. In the following sections, we will discuss specific scenarios in which the presence of a pathogenic variant itself could potentially have an adverse effect on a SMs’ career. In contrast to results from diagnostic testing or familial cascade screening, secondary findings and associated implications may be unexpected in individuals previously presumed healthy. Management recommendations for those that harbor a pathogenic variant regardless of phenotype are relatively new, continue to be refined, and may change with further research. Some of these management recommendations, such as exercise restriction, may be incompatible with military active duty. We discuss Malignant Hyperthermia (MH) and Arrhythmogenic Right Ventricular Cardiomyopathy/Dysplasia (ARVC/D) to illustrate cases in which management recommendations may impact the ability to continue to serve in the military for those found to have a pathogenic variant, with the acknowledgment that career impact decisions should be made by the clinical care team with respect to each individual.

## 5 MALIGNANT HYPERTHERMIA

Malignant Hyperthermia is a disorder characterized by uncontrolled calcium release from the sarcoplasmic reticulum of skeletal muscle, usually in response to inhaled general anesthetics (excluding nitrous oxide) and/or succinylcholine. In addition, environmental factors such as exercise or high ambient temperatures may also precipitate crises in some individuals (Potts et al., 2014). Clinically, the syndrome is characterized by hyperthermia, tachycardia, muscle rigidity, rhabdomyolysis, hyperkalemia, and lactic acidosis. These reactions may lead to morbidity and mortality (Lee, McGlinch, McGlinch, & Capacchione, 2017). Treatment with dantrolene, cooling, and supportive measures is life‐saving but early identification and diagnosis are imperative (Riazi, Kraeva, & Hopkins, 2018). Affected individuals benefit from preventive measures that include avoidance of precipitating agents and in some cases, avoidance of extremes of heat. Due to the risk of death in MHS patients exposed to triggering agents and/or heat, personal history of MH is a disqualifying condition for admission into the armed services and those diagnosed later in their military career may be medically discharged (Stanley, 2001). It is estimated that the prevalence of genetic variants predisposing to MH is as high as 1:2,000 (Monnier et al., 2002). There are currently two genes associated with MH on the ACMG/AMP 59 gene list recommended to be returned to those undergoing genomic sequencing—*RYR1 *and *CACNA1S*.

## 6 ARRHYTHMOGENIC RIGHT VENTRICULAR CARDIOMYOPATHY/DYSPLASIA

Arrhythmogenic Right Ventricular Cardiomyopathy/Dysplasia (ARVC/D) is an inherited cardiac condition that causes progressive fibro‐fatty replacement of (mainly) the right ventricular myocardium, predisposing affected individuals to ventricular tachycardia and sudden death. Patients usually present in young adulthood with palpitations and syncope; however, sometimes sudden cardiac death may be the initial presentation. Current research suggests that penetrance is significantly higher for those who exercise compared to those who do not (Sawant et al., 2013). Given this finding, both those with a clinical diagnosis and those identified to have a pathogenic variant known to be associated with ARVC/D are strongly discouraged from participating in frequent vigorous endurance exercise. The prevalence of ARVC/D is estimated to be 1:1,000–1:1,250 (Olfson et al., 2015). There are five genes associated with ARVC/D‐ *PKP2, DSP, DSC2, TMEM43, *and *DSG2*, included on the ACMG/AMP 59 gene list recommended to be returned to those undergoing genomic sequencing.

## 7 OTHER CONDITIONS

Akin to the medical management recommendations for MH and ARVC/D, other conditions represented on the ACMG/AMP 59 gene list and further expanded secondary findings lists may impact continued service. It is important to consider the medical management recommendations for individuals found to have pathogenic variants related to disease risk and how these management recommendations may or may not impact service ability. It is also important to note that currently there are some conflicting medical management recommendations for certain diseases with respect to genotype in the absence of phenotype. This makes creating broad policies regarding pathogenic variants in these genes and related military service impractical at this time. Further research is needed to clarify risk, penetrance, expressivity, and medical management recommendations for individuals with a pathogenic variant in the absence of clinical phenotype.

## 8 CONCLUSION

Similar to research and clinical testing in civilian setting, there are risks and benefits to genomic sequencing for the SM. Benefits to disclosing genomic sequencing results, including secondary findings, can include changes in healthcare management and lifestyle that could potentially be life‐saving for an individual. In addition, these findings may also benefit the health care of family members and may theoretically benefit the SM's unit (e.g., if the service member has an event requiring medical evacuation, this may compromise the position of the unit and/or prevent critical assets from being used for others who may need it). For the military population, however, while the return of such findings may positively impact health, it may also impact career, as some medical management recommendations for those with a pathogenic variant, even without associated phenotype, may not be compatible with military service. The reason for this is the protection of the SM and the mission since military service is associated with risk factors for these severe diseases including frequent, strenuous physical activity, and potential deployment to austere environments. As our understanding of the complete phenotypic spectrum, penetrance, and expressivity of these genes and conditions advances, we may be in a better position to craft nuanced policies with regard to ability to serve and potential duty limitations. Further genomic sequencing studies that examine these critical aspects are needed to create better medical management guidelines for individuals with pathogenic variants associated with disease risk, in the absence of clinical phenotype. In the meantime, we strongly encourage all researchers and clinicians working with SMs to discuss both the risks and benefits of genomic sequencing in order to ensure proper informed consent.

## CONFLICT OF INTEREST

The authors report no conflict of interest. The views expressed in this article are those of the author and do not reflect the official policy of the Department of Army/Navy/Air Force, Department of Defense, or U.S. Government.
